# Comparison of First Childbirth Characteristics between Elite Judo Athletes and Non-Athletes: The Preliminary Retrospective Case–Control Study

**DOI:** 10.3390/ijerph192013218

**Published:** 2022-10-14

**Authors:** Anna Kuczera, Agnieszka Opala-Berdzik, Jitka Malá, Marcin Sodowski, Daria Chmielewska

**Affiliations:** 1 Students Scientific Association on the Analysis of the Influence of Pregnancy on the Musculoskeletal System, Physiotherapy Faculty, The Jerzy Kukuczka Academy of Physical Education, 40-065 Katowice, Poland; 2 Department of Physiotherapy in Internal Diseases, Institute of Physiotherapy and Health Sciences, The Jerzy Kukuczka Academy of Physical Education, 40-065 Katowice, Poland; 3 Physiotherapy Department, Faculty of Physical Education and Sport, Charles University, 121 08 Prague, Czech Republic; 4 Gynecology and Obstetrics Ward, City Hospital in Siemianowice Śląskie, 41-100 Siemianowice Slaskie, Poland; 5 Electromyography and Pelvic Floor Muscles Laboratory, Department of Physical Medicine, Institute of Physiotherapy and Health Sciences, The Jerzy Kukuczka Academy of Physical Education, 40-065 Katowice, Poland

**Keywords:** elite judo athlete, childbirth outcomes, labor, delivery

## Abstract

It has been speculated that elite athletes are more likely to have obstetric interventions during labor and delivery. So far, the impact of many years of competitive sports participation on childbirth characteristics has not been well-established. This preliminary retrospective case–control study aimed to determine whether the first labors of elite judo competitors required obstetric interventions more frequently and were longer than those of non-athletes. The study comprised 32 parous women: 16 elite judo athletes and 16 non-athletes. Women were included if they had access to the following obstetric data (from their first childbirth hospital discharge reports and/or first child’s health record books): induction and augmentation of labor, mode of delivery, the duration of labor and its second stage, episiotomy, perineal tear, and the neonate’s Apgar score. Electronic surveys were completed concerning childbirth characteristics, level of “eliteness” (judo athletes), and recreational physical activity (non-athletes). The statistical analysis showed no significant between-group differences in any of the first childbirth outcomes under analysis. The preliminary results indicate elite judo practice before first pregnancy had no negative impact on the rates of labor induction and augmentation, delivery mode, duration of labor, the rates of episiotomy and perineal tear, and the neonate’s Apgar score. Due to the small sample size, the conclusions should be considered with caution.

## 1. Introduction

Judo is one of the Olympic combat disciplines; the characteristics of elite judo competitors are, among others, excellent muscle strength, power, and endurance [1]. With a high-level performance, the athletes display superior trunk and core stability [2]. As the pelvic floor muscles belong to the core muscle group, it can be assumed that elite judo athletes exhibit increased strength and, in consequence, hypertrophy of these muscles. Although similar studies on judo competitors are lacking, magnetic resonance and ultrasound imaging studies demonstrated increased cross-sectional areas and diameters of the levator ani muscles in groups of nulliparous elite athletes that comprised runners, netball and basketball players, dancers, aerobics competitors, squash and tennis players, and gymnasts. Some of these athletes reported that their training routines included core stability training involving voluntary contractions of the transversus abdominis and pelvic floor muscles [3,4]. However, another study that used a perineometer demonstrated that female athletes (volleyball, handball, and basketball players) had lower perineal pressure during maximum isometric contractions of the pelvic floor muscles than non-athletes. Concomitantly, decreased perineal pressure correlated with increased symptoms of urinary incontinence and pelvic floor dysfunction [5].

Although a recent review with meta-analysis classified judo as having a high impact on the pelvic floor (similar to gymnastics, ballet, aerobics, jump sports, soccer, basketball, handball, and volleyball) [6], different rates of urinary incontinence were demonstrated among nulliparous athletes depending on sport discipline. In total, 89% of 9 artistic and trampoline gymnasts, 50% of 26 swimmers, 43.5% of 23 volleyball players, and 44.4% of 9 judo players reported incontinence (vs. 27% of 96 age-matched non-athletes) [7], indicating the impact of elite athletic training on pelvic floor structure and function is discipline-specific. Therefore, it appears important that studies on athletes practicing high-impact sports also involve separate assessments of single discipline subgroups. Since the mechanisms triggering increases in intrabdominal pressure and consequent effects on pelvic floor structures may be different depending on the discipline (the training may focus on frequent jumps, strenuous activities leading to Valsalva maneuvers, or long-lasting physical efforts), it appears that the recommendations for pelvic floor muscle exercises for the athletes should be sport-specific.

It should be noted though that vaginal delivery requires the adequate stretching ability of the levator ani muscle during the second stage of labor [8]. Failure of the levator ani to relax may be associated with a longer duration of the second stage of labor and a higher risk of intrapartum cesarean and instrumental vaginal deliveries [9]. A study with models of the female athlete and non-athlete pelvic floor and simulation of the fetal head descending through the pelvic floor as in vaginal delivery showed that athletes might require greater force to achieve delivery compared to non-athletes [10]. This may suggest that, during spontaneous pushing in the second stage of labor, athletes need to increase the intra-abdominal pressure to a greater extent than non-athletes. Pushing with closed glottis (the Valsalva maneuver) is associated with a maximal increase in the intra-abdominal pressure and reflex contraction of the levator ani muscle which may lead to a prolonged second stage of labor [11,12]. The pattern of spontaneous pushing in elite athletes should therefore be investigated.

According to a recent systematic review and meta-analysis, the impact of many years of sports practice at a competitive level on childbirth outcomes has not been well-established [13]. The scant literature in this area suggests there are no significant differences between elite athletes and controls concerning delivery mode [14], preterm birth, cesarean delivery rate, low neonate birth weight, [15], the length of the first and second stages of labor, intrapartum cesarean delivery rates, and perineal tears [16].

The majority of research into the impact of physical activity on pregnancy and childbirth outcomes has focused on regular recreational-level exercise during pregnancy. This type of physical activity is associated with decreased rates of excessive gestational weight gain, gestational diabetes mellitus [17], gestational hypertensive disorders, preterm [18] and cesarean delivery [19,20], and an increased rate of vaginal delivery [21]. It has also been shown that regular prenatal exercise at a recreational level has no negative effect on the duration of the second stage of labor [22,23], the total duration of labor [20], the rate of labor induction, elective and intrapartum cesarean delivery, episiotomy, instrumental vaginal delivery, third- and fourth-degree perineal tear, neonate birth weight [22] and Apgar score [20]. According to the 2020 exercise guidelines of the American College of Obstetricians and Gynecologists, pregnant women are encouraged to perform aerobic and strengthening exercises of moderate intensity for at least 150 min per week [24].

However, there is still a need to investigate childbirth characteristics in women who have long-term sports practice with higher training loads. The evidence summary produced by experts of the International Olympic Committee (IOC) leaves many unanswered questions concerning perinatal outcomes in elite athletes [25].

This preliminary retrospective study aimed to determine the association between engagement in elite athletic training before first pregnancy (elite judo athletes vs. non-athletes) and labor induction and augmentation, delivery mode, episiotomies, and perineal tears during first childbirths. Another aim was to establish whether there were differences in the length of the whole labor process and its second stage, and the neonates’ Apgar scores between these two groups. Considering the available knowledge and premises on the possible impact of strenuous long-term sports training on childbirth outcomes, we hypothesized that there might be higher rates of childbirths with obstetric interventions and longer labors in elite judo athletes. Conducting this preliminary study facilitated the verification of the capability of the study design, recruitment criteria and procedures, and analyzed variables to be used in a larger study.

## 2. Materials and Methods

Forty healthy paras were recruited in this retrospective case–control study. The recruitment took place between June and September 2021. The cases were 21 elite judo athletes, and the controls were 17 non-athletes. For the judo group, women were recruited by the first author (A.K.) who, as an elite judo competitor had met the athletes at the National Team and international tournaments. For the non-athlete group, the women were recruited among colleagues of the first author (A.K.). The recruitment took place through phone calls and social media.

Inclusion criteria for the group of elite judo athletes were a history of performance achievements at national and international championships. The criterion for inclusion in the non-athlete group was no history of athletic training at a competitive level. Additionally, to match the age, the inclusion criteria for the non-athlete controls was the age range of the judo group. The women were included in both groups if they had a history of one or first singleton vaginal or intrapartum cesarean delivery, access to first childbirth hospital discharge reports and/or first child’s health books, and very good knowledge of English or Polish. The exclusion criterion for both groups was a history of planned cesarean delivery. At the level of verification of completed surveys, the exclusion criterion was pre-pregnancy body mass index (BMI) ≥30 because obesity may influence childbirth outcomes [26]. The data of elite judo athletes were also excluded if the period from the withdrawal from regular judo-specific high-intensity training sessions to the beginning of the first pregnancy exceeded 12 months.

The study was carried out using an electronic survey created in Polish and English. The link to the survey was made available to the study participants in direct messages on social media. The women were asked to fill out the surveys on their obstetric data using information included in their first childbirth hospital discharge reports and/or their first child’s health books. The following obstetric aspects of first childbirth were addressed in the survey: the induction and/or augmentation of labor with oxytocin, mode of delivery (vaginal, intrapartum cesarean, or assisted vaginal); the length of the whole labor process and its second stage, perineal tear, episiotomy, and the neonate’s Apgar score (in case of vaginal delivery). Additionally, elite judo athletes responded to questions about their career achievements (Table 1). The level of eliteness was determined according to the classification proposed by Swann et al. [27]. Non-athlete women completed a survey on their recreational physical activity levels during pregnancy. They were asked about the duration, frequency, and type of their physical activity in the second and third trimesters of pregnancy, and whether they performed pelvic floor muscle exercises. The physical activity was considered regular if it was performed for at least 30 min, 2–3 times a week (Table 2).

The following are definitions of the obstetric terms used:

- Labor induction: artificial prompting of the uterus to contract before labor begins on its own. It may be implemented due to various medical indications related to maternal and fetal health status [28].
- Labor augmentation: application of intravenous oxytocin to hasten labor and shorten the time to delivery (used in longer labors or less frequent uterine contractions) [29].
- Intrapartum (emergency) cesarean delivery is performed after labor onset [30].
- The first stage of labor: starts with the onset of regular uterine contractions and ends with full cervical dilation to 10 cm. The absence of cervical change for greater than four hours in the presence of adequate contractions or six hours with inadequate contractions is considered the arrest of labor and may require clinical intervention [31].
- The second stage of labor is the time between complete dilation of the cervix (about 10 cm) and neonate delivery. It typically lasts less than three hours in nulliparas and less than two hours in multiparas (in labors with neuraxial anesthesia it is longer) [31].
- assisted (instrumental) vaginal deliveries mainly involve the use of forceps or a vacuum cup to expedite delivery, usually during delays in the second stage of labor when it is impossible or unsafe to perform a cesarean section [32].
- Perineal tears differ in degree of laceration involving: (1) vaginal mucosa or perineal skin only, (2) the perineal muscles, (3) the anal sphincter muscles, and (4) the anal epithelium [33].
- Episiotomy is a surgical incision of the perineum and the posterior vaginal wall aiming to widen the vaginal outlet, facilitate delivery, and reduce the rates of severe perineal tears; however, recent research has not supported the theory that it prevents pelvic floor trauma [33].
- The Apgar scale is a tool used to assess a newborn’s clinical status. Apgar scores are reported at 1 and 5 min after birth for all infants. The acronym stands for the following characteristics in a newborn: Appearance—skin color, Pulse—heart rate, Grimace—reflexes, Activity—muscle tone, and Respiration—breathing. It does not predict individual neonatal mortality or neurologic outcome [34].

A Mann–Whitney U test was used to compare subject characteristics concerning first pregnancy and childbirth: age and gestational age at childbirth, body height, pre-pregnancy/pre-labor body mass and BMI, neonate birth weight, the length of the whole labor process and its second stage, and the neonate’s Apgar scores (for vaginal deliveries) between cases and controls.

Chi-squared (χ^2^) test with Yates correction was used to determine relationships between engagement in elite athletic training (elite judo athletes/non-athletes) and labor induction and augmentation (the values of the expected cells were 5 or more in at least 80% of the cells, and no cell had an expected value of less than one). χ^2^ test was used to determine relationships between engagement in elite athletic training (elite judo athletes/non-athletes) and mode of delivery, and perineal tears. Fisher’s exact test was used to determine the relationship between engagement in elite athletic training (elite judo athletes/non-athletes) and episiotomies (the value of expected call was less than 5) [35]. The level of significance was set at *p* < 0.05. The rates of obstetric procedures/perinatal complications in both groups were calculated as percentages. Analyses were performed using Statistica v.13 (TIBCO Software, Palo Alto, CA, USA).

## 3. Results

In total, six surveys were excluded from the analysis due to not meeting eligibility criteria at the level of survey responses verification. Of the case subjects, in five the period from the withdrawal from regular judo-specific high-intensity training sessions to the first pregnancy exceeded 12 months. One control subject did not meet the BMI eligibility criterion (had BMI > 30). Therefore, a total of thirty-two surveys were analyzed, i.e., 16 out of 21 elite judo athlete surveys and 16 out of 17 non-athlete surveys.

At the time of completing the survey on first childbirth characteristics, of the 16 female judo athletes, 2 had a history of two deliveries and the remaining 14 were primiparas. All 16 non-athlete controls were primiparas. The judo athletes were representatives of six countries. There were 13 athletes from Europe (11 from Poland and 2 from other countries), 2 from Africa, and 1 from North America. All the non-athletes were of Polish nationality. In all study participants, the period between first delivery and response to the survey ranged from one month to five years.

The elite judo athletes were National and World Cup Championships competitors/ medalists as well as Continental, World, and Olympic Championships competitors/medalists. Table 1 presents categories of “eliteness” in judo athletes based on their best carrier achievements (six presenting competitive, three successful, and seven world-class categories) [24] and the periods between their withdrawal from regular judo-specific high-intensity training sessions and the beginning of their first pregnancies.

Of the 16 athletes, 10 stopped participating in routine twice-a-day high-intensity training sessions and competitions just before getting pregnant (0–2 months) while 6 quit training between 5 and 12 months before getting pregnant. However, they continued daily general body conditioning exercises (moderate to high intensity) before and during pregnancy (information based on personal communication).

Table 2 presents the level of physical activity during pregnancy in 16 non-athletes.

The Mann–Whitney U test showed no significant differences in obstetric characteristics concerning first pregnancies and childbirths between 16 elite judo athletes and 16 non-athletes (*p* > 0.05, Table 3).

**Figure 1:**
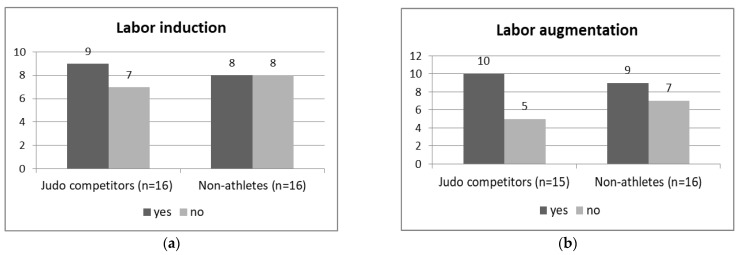
The numbers of first labor (**a**) induction [χ^2^ with Yates correction (1) = 0, *p* = 1], and (**b**) augmentation [χ^2^ with Yates correction (1) = 0.05, *p* = 0.82] in elite judo athletes and non-athlete controls.

Nine (56.25%) of the sixteen elite judo athletes and eight (50%) of sixteen non-athletes had first labors induced by oxytocin administration. One judo athlete did not respond to a question regarding intrapartum labor augmentation. Ten (66.7%) of the remaining fifteen judo athletes and nine (56.25%) of sixteen non-athletes had their labors stimulated by oxytocin. The χ^2^ test with Yates correction indicates no significant relationships between engagement in elite athletic training before pregnancy (elite judo athletes/non-athletes) and labor induction or augmentation (*p* > 0.05, Figure 1a,b).

**Figure 2:**
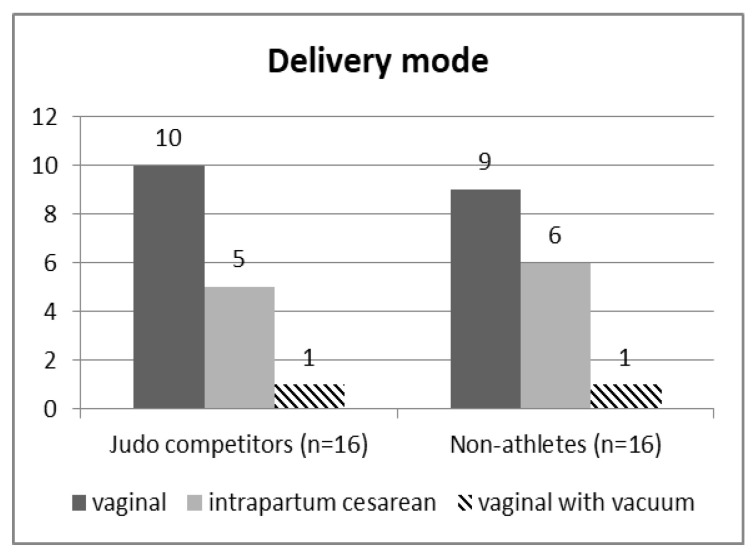
The numbers of vaginal, intrapartum cesarean, and vacuum-assisted vaginal deliveries in elite judo athletes and non-athlete controls [χ^2^ (2) = 0.14, *p* = 0.93].

Of sixteen elite judo athletes, ten had vaginal (62.5%), five intrapartum cesarean (31.25%) and one (6.25%) vacuum-assisted vaginal deliveries. Of the sixteen non-athletes, nine (56.25%) had vaginal, six (37.5%) intrapartum cesarean, and one (6.25%) vacuum extraction deliveries. According to the results of the χ^2^ test, there was no significant relationship between engagement in elite athletic training before pregnancy (elite judo athletes/non-athletes) and mode of delivery (*p* > 0.05, Figure 2).

Further results concern women who delivered vaginally: 11 elite judo athletes and 10 non-athletes. The Mann–Whitney U test revealed no significant differences between the two groups regarding the length of the whole labor process and its second stage, and the condition of the newborns assessed at 1 and 5 min after birth using the Apgar scale (*p* > 0.05; Table 4).

Of the eleven elite judo athletes, five (45.5%) experienced perineal tears; three (27.3%) and two (18.2%) had grade I and grade II perineal tears, respectively. Of the ten non-athletes, two (20%) experienced perineal tears that were both grade II. The χ^2^ test showed no relationship between engagement in elite athletic training before pregnancy (elite judo athletes/non-athletes) and perineal tears.

**Figure 3:**
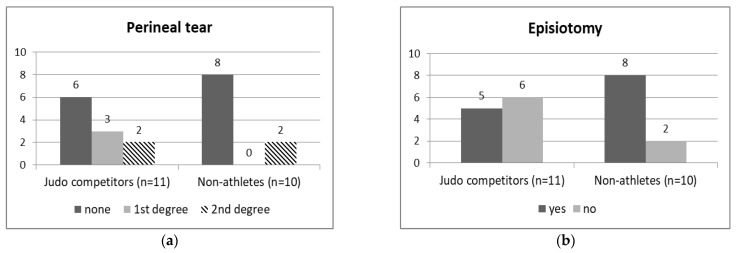
The numbers of (**a**) perineal tears (χ^2^ = 4.39, *p* = 0.11) and (**b**) episiotomies (Fisher’s exact test: *p* = 0.11) in in elite judo athletes and non-athlete controls.

Episiotomy was performed in five (45.5%) of the eleven judo athletes and eight (80%) of the ten non-athletes. Fisher’s exact test indicated no relationship between engagement in elite athletic training before pregnancy (elite judo athletes/non-athletes) and episiotomies (Figure 3a,b).

To eliminate the possible impact of a longer period (5–12 months) between taking a career break and first pregnancy on delivery outcomes, additional statistical analyses were performed to compare 10 judo athletes (who quit the sport 0–2 months before getting pregnant) with 16 non-athletes. The analyses demonstrated no significant differences in age and gestational age at childbirth, body height, pre-pregnancy and pre-labor body mass and BMI, and neonate birth weight between the two groups. There were no relationships between engagement in elite athletic training before pregnancy (elite judo athletes/non-athletes) and labor induction or augmentation, and mode of delivery. With regard to natural births, no differences were found in the length of the whole labor process and its second stage, and newborns’ Apgar scores at 1 and 5 min after birth; there were also no relationships between elite athletic training before pregnancy (elite judo athletes/non-athletes) and perineal tears or episiotomies (*p* > 0.05; Table 5).

## 4. Discussion

This preliminary study aimed to determine first childbirth characteristics including labor induction and augmentation, delivery mode (vaginal, intrapartum cesarean, or assisted vaginal), length of the whole labor process and its second stage, perineal tear, episiotomy, and the neonate’s Apgar score (vaginal delivery) in elite judo athletes, and to compare these variables to non-athlete counterparts. It was hypothesized that many years of judo practicing at an elite level might lead to higher rates of childbirth requiring obstetric interventions and longer births. However, our preliminary results based on a sample of 32 women indicate that elite judo athletes and non-athlete controls had similar childbirth outcomes. This suggests long-term high-intensity judo training had no adverse effect on the course of the first labor and delivery. Although these findings stand in contrast to the existing premises suggesting that elite athletes might experience more difficult deliveries [3,4,10], they are consistent with the conclusions of a recent systematic review and meta-analysis suggesting that competitive sports training does not increase the risk of perinatal complications. The authors of the review pointed out though that the existing evidence on pregnancy and childbirth outcomes in elite athletes was limited and of “very low” certainty due to the observational nature of the studies [13].

Our preliminary study suggests that long-term high-intensity judo training before first pregnancy did not lead to an increase in the number of first labor induction or augmentation. This may suggest that elite judo athletes were not at higher risk of insufficient uterine contractions for beginning or continuing labor. To the authors’ best knowledge, the induction and augmentation of labor have not been so far compared between elite athletes and non-athletes. However, it was demonstrated that regular recreational-level physical activity did not influence labor induction [22].

The present study shows no association between engagement in elite judo practice before first pregnancy and a higher number of intrapartum cesarean deliveries. However, this preliminary study does not provide information on what were the indications for this mode of delivery in both study groups. Because the intrapartum cesarean delivery may be related to various risk factors such as cervical dilation at admission, BMI, oxytocin, chorioamnionitis, epidurals, labor length, or birth weight [30], it should be further investigated whether indications for intrapartum cesarean deliveries differ between elite judo athletes and non-athletes.

Our finding of no association between judo practice at the highest level before first pregnancy and a higher number of intrapartum cesarean deliveries is consistent with previous reports indicating no differences in delivery mode/rates of cesarean or intrapartum cesarean delivery between elite athletes and controls [14,15,16]. However, it should be noted that the controls in those studies were either active at the recreational level during pregnancy [15,16] or presented mixed (from no to recreational-level) exercise participation [14]. In our study, of sixteen controls, four exercised regularly at the recreational level, and the remaining twelve exercised irregularly or did not exercise at all during pregnancy. Because randomized control trials showed a decrease in cesarean delivery rate in women performing aerobic exercises three to four days a week during pregnancy compared to non-exercisers [19,20], further investigation should also focus on determining whether elite sports practice contributes to a decrease in cesarean section rates compared to lack of engagement in antenatal recreational physical activity.

Regarding the duration of the first labor, the preliminary results indicate that many years of high-impact judo practice before pregnancy did not lead to the increase in the length of the entire labor and its pushing phase. This may suggest that potentially different parameters of the abdominal and pelvic floor muscles in judo athletes did not have a negative impact on the effectiveness of their pushing during the second stage of labor. Our results are in agreement with another study suggesting that women involved in competitive sports training did not experience longer first and second stages of labor compared to recreationally active controls [16]. However, a shorter duration of active labor (second stage of labor) was recently reported in non-athlete women with higher levels of daily physical activity in pregnancy assessed with the Kaiser Physical Activity Survey [36]. Additionally, the results of systematic reviews and meta-analyses indicate that antenatal exercise (not related to competitive sports), as well as pelvic floor muscle exercises, may reduce the duration of the second stage of labor [37,38]. In our study, of the sixteen non-athlete controls, four exercised recreationally and three performed pelvic floor muscle exercises during pregnancy; therefore, further research should compare elite athletes to three separate groups of non-athletes: non-exercisers, recreational exercisers, and pelvic floor muscle exercisers to verify the effect of elite athletic training on labor duration.

Our preliminary results indicate no association between engagement in elite judo practice before first pregnancy and the incidence of perineal tears during first deliveries. In both groups, there were only grade I and grade II tears that do not lead to anal sphincter injury [33]. This suggests that the ability of the pelvic floor muscles to stretch during childbirth in judo athletes and non-athletes was similar. Our results are consistent with the conclusion of Sigurdardottir et al. [16], who did not observe any relation between practicing sports at the elite level and the incidence of severe perineal tears during childbirth.

The association between engagement in elite judo practice and episiotomies also turned out to be non-significant in our study. This suggests that in the judo athletes there was no greater need to perform episiotomy for widening the vaginal outlet and facilitating delivery than in the non-athletes. We have not found other studies addressing this obstetric outcome in elite athletes. A study on women engaging in general exercise before and during pregnancy suggested no negative effect of that type of physical activity on episiotomy rate [22].

The present study also attempted to compare the condition of newborns (assessed by the Apgar scale) of judo athlete and non-athlete mothers who delivered vaginally. The results indicate similar Apgar scores at 1 and 5 min after birth in the two groups suggesting no higher risk of poorer neonate clinical status in case of vaginal delivery of elite judo athletes. To the authors’ best knowledge, there are no publications on this subject concerning women involved in competitive sports at the elite level. There is only one study on recreational prenatal exercise showing no differences in newborns’ Apgar scores between physically active and sedentary mothers [20].

The strength of the present study is the inclusion of a selected group of elite judo athletes of the highest competitive level who were National and World Cup Championships competitors and medalists as well as Continental, World, and Olympic Championships competitors and medalists, and who relatively recently gave birth to the first child. The athletes had been involved in their regular high-intensity training regimens for an average of up to 4 months before becoming pregnant. However, the recruitment of such a research group is challenging. The first author of the study, as an elite judo competitor, used her personal contacts to recruit participants.

### Study Limitations and Indications for Further Research

This preliminary study is limited by its small sample size and indirect retrospective nature of obstetric data collection. Therefore, all conclusions should be considered with caution. Further research should be based on direct access to participants’ data from the obstetric units. In addition to obstetric outcomes that were analyzed in this study, more detailed information should be collected such as indications for intrapartum cesarean and assisted (instrumental) vaginal deliveries. Assessment of other variables including miscarriages, fetal growth, newborn’s birth weight, preterm birth, epidural anesthesia, and elective cesarean delivery may be also considered [25].

Another limitation of this preliminary study is the fact that of the 16 elite judo athletes, 10 stopped participating in routine twice-a-day high-intensity training sessions just before getting pregnant (0–2 months), while 6 quit their sports career between 5 and 12 months before conception. To eliminate the possible impact of a longer break between very high-intensity training and pregnancy, data of 10 judo athletes (with a period of 0–2 months from quitting their career to conception) and 16 non-athletes were also compared. Although there were non-significant between-group differences, our results should be verified on a larger sample.

Furthermore, the data on elite athletes’ physical activity after withdrawing from regular training sessions and during pregnancy were limited to information that its nature changed to general body conditioning training, the frequency was reduced from twice to once a day, and intensity to moderate-to-high. In further research, a survey should be used to collect more detailed information on the elite athletes’ physical activity characteristics as well as their core stability/pelvic floor muscle exercises after withdrawing from their routine training sessions and during pregnancy.

Another limitation of this preliminary study is that the included elite judo athletes were representatives of different countries (11 from one country, and 5 from other countries) while the control group comprised women from a single country (the same as 11 judo athletes). As a more extensive literature search revealed that ethnicity/race as well as country-specific obstetric guidelines may influence delivery outcomes [39,40,41,42,43,44,45], further research on a larger sample involving athletes from various countries should recruit a control group from the corresponding countries. Such recruitment could be carried out by an international multi-center study.

When considering our results and conclusions, it should be noted that elite athletes were compared to non-athletes of whom four were regular recreational exercisers, three pelvic floor muscle exercisers, and nine non-exercisers during pregnancy. Because regular recreational physical activity and pelvic floor muscle training may impact delivery outcomes [19,20,37,38], further research should compare delivery outcomes in elite athletes to separate groups of non-athletes who regularly exercised recreationally, performed pelvic floor muscle exercises, and did not engage in any exercises before and during pregnancy.

This preliminary study indicates that devising a high-quality study on the impact of elite sports training on delivery outcomes might be challenging. It appears a systemic solution might be helpful to carry out research on a larger sample. The development of specific tools for the IOC, such as post-career and pregnancy physical activity questionnaires to be given to the athletes, as well as delivery outcome form to be handed out by the athletes during admission to the delivery rooms and filled out by the obstetric staff would allow the prospective collection of physical activity and obstetric data.

## 5. Conclusions

This preliminary study indicates no negative effect of elite judo practice before first pregnancy on childbirth outcomes such as the rates of labor induction and augmentation, mode of delivery, the total duration of labor and its second stage, the rates of perineal tear, episiotomy, and the neonate’s Apgar score.

## Figures and Tables

**Table 1:** Categories of judo athletes “eliteness” * and the periods between their withdrawal from regular judo-specific high-intensity training sessions and the beginning of their first pregnancy.

ID	Category of “Eliteness”	The Period from Career Withdrawal to Conception (Months)
1	Successful	0
2	World-class	2
3	World-class	0
3	Competitive	8
5	Competitive	0
6	Competitive	0
7	Competitive	5
8	Competitive	12
9	World-class	1
10	Competitive	0
11	Successful	6
12	Successful	12
13	World-class	2
14	World-class	11
15	World-class	0
16	World-class	2

* Competitive-elite athlete regularly competes at the highest level (e.g., top divisions/leagues, the Olympic Games) without any achievements; successful-elite athlete competes at the highest level with some (infrequent) success (e.g., winning an event or a medal); world-class elite athlete experiences sustained success at the highest level, with repeated wins over a prolonged time (e.g., winning gold medals in consecutive Olympics, or major competitive victories over many seasons) [27].

**Table 2:** Regular prenatal, individual or organized, recreational physical activities (at least 30 min 2–3× week) in non-athletes ^1^.

ID	Regular Physical Activity	2nd Trimester Physical Activity Duration/Frequency	3rd Trimester Physical Activity Duration/Frequency	Regular PFM * Exercises (3–5× Wk)	Trimester, Frequency, Series, Repetitions of PFM Exercises	Type of Regular Physical Activity
1	No	-	-	No	-	-
2	No	-	-	No	-	-
3	No	-	-	No	-	-
4	No	-	-	No	-	-
5	Yes	Daily, 60 min	Daily, 60 min	No	-	Walking
6	Yes	2× wk, 30–40 min	1–2× wk, 30 min	No	-	Swimming
7	No	-	-	No	-	-
8	Yes	5× wk, 90 min	5× wk, 90 min	No	-	Fitness, walking
9	Yes	Daily, 30 min	2–3× wk, 30min	No	-	Joga, pilates, walking
10	No	-	-	Yes	2nd and 3rd trimesters, 3–5× wk, 10× 2 series	-
11	No	-	-	No	-	-
12	No	-	-	No	-	-
13	No	-	-	Yes	3rd trimester, 4× wk, 20–30× 2 series	-
14	No	-	-	No	-	-
15	No	-	-	Yes	3rd trimester, 5× wk, 25× 4 series	-
16	No	-	-	No	-	-

^1^ the remaining female non-athletes exercised irregularly or did not exercise at all; * PFM—pelvic floor muscles.

**Table 3:** Obstetric characteristics of elite judo athletes and non-athletes related to their first pregnancy and childbirth *.

	Judo Athletes (*n* = 16)	Non-Athletes (*n* = 16)	U	*p*
**Age at childbirth (years)**	26.3 ± 4.1 (20–34)	26.4 ± 2.5 (22–31)	125.5	0.93
**Gestational age at childbirth (weeks)**	39.7 ± 2.0 (35–43)	39.5 ± 2.0 (35–42)	123	0.87
**Body height (cm)**	167.6 ± 7.8 (153–187)	167.4 ± 5.0 (158–178)	126.5	0.96
**Pre-pregnancy body mass (kg)**	63.4 ± 9.3 (47–78)	63.8 ± 8.7 (54–81)	127	0.99
**Pre-labor body mass (kg)**	83.5 ± 11.0 (60–103)	79.0 ± 11.0 (63–110)	86.5	0.12
**Pre-pregnancy BMI**	22.5 ± 2.4 (19.0–27.0)	22.8 ± 3.1 (18.6–29.7)	124	0.90
**Pre-labor BMI**	29.7 ± 3.3 (23.7–35.6)	28.2 ± 4.1 (22.9–40.4)	87	0.13
**Neonate birth weight (g)**	3301.6 ± 465.6 (2400–4180)	3313.4 ± 454.5 (2525–4150)	124.5	0.90
**The period from career withdrawal to conception (months)**	3.8 ± 4.6 (0–12)	-	-	-

* Data are shown as means ± standard deviations (ranges); Mann–Whitney U test.

**Table 4:** Duration of labor and the neonate’s Apgar score in elite judo athletes and non-athletes with vaginal deliveries *.

	Judo Athletes (*n* = 11)	Non-Athletes (*n* = 10)	U	*p*
**Whole labor (min)**	664.1 ± 667.9 (120–2160)	507.3 ± 512.0 (90–1740)	51	0.81
**2nd stage of labor (min)**	45.5 ± 48.3 (5–180)	46.8 ± 38.1 (10–128)	52.5	0.86
**1st min Apgar score**	7.6 ± 3.6 (0–10)	9.5 ± 0.7 (8–10)	36.5	0.2
**5th min Apgar score**	8.8 ± 2.2 (4–10)	9.6 ± 0.7 (8–10)	52.5	0.86

* Data are shown as means ± SD (ranges); Mann–Whitney U test.

**Table 5:** Results of comparisons of obstetric characteristics related to first pregnancy and childbirth between elite judo athletes (who stopped their routine training sessions between 0 and 2 months before pregnancy) and non-athletes.

	Judo Athletes	Non-Athletes	Statistical Analyses	
	*n*	*n*	U/χ^2^/FE *	*p*
**Age at childbirth**	10	16	U = 72.5	0.7
**Gestational age at childbirth**	10	16	U = 72.5	0.7
**Body height**	10	16	U = 67	0.52
**Pre-pregnancy body mass**	10	16	U = 71.5	0.66
**Pre-labor body mass**	10	16	U = 52	0.15
**Pre-pregnancy BMI**	10	16	U = 78	0.94
**Pre-labor BMI**	10	16	U = 63	0.39
**Neonate birth weight**	10	16	U = 64.5	0.42
**Labor induction (yes/no)**	10	16	χ^2^ = 0.0	1.0
**Labor augmentation (yes/no)**	10	16	FE	0.46
VD **/intrapartum CC ***	10	16	FE	0.4
**Whole labor duration**	5	10	U = 23.5	0.86
**2nd stage labor duration**	5	10	U = 23.5	0.86
1st min Apgar score (VD **)	5	10	U = 19	0.51
5th min Apgar score (VD **)	5	10	U = 24	0.95
Episiotomy (yes/no; VD **)	5	10	FE	0.4
Perineal tear (yes/no; VD **)	5	10	FE	0.41

* U—Mann–Whitney U test; χ^2^—Chi^2^ test FE—Fisher’s exact test; ** VD—vaginal delivery, *** CC—cesarean section.

## Data Availability

Data supporting reported results can be obtained from the corresponding author upon reasonable request.

## References

[1] Franchini E., Del Vecchio F.B., Matsushigue K.A., Artioli G.G. (2011). Physiological profiles of elite judo athletes. Sports Med..

[2] Barbado D., Lopez-Valenciano A., Juan-Recio C., Montero-Carretero C., van Dieën J.H., Vera-Garcia F.J. (2016). Trunk Stability, Trunk Strength and Sport Performance Level in Judo. PLoS ONE.

[3] Kruger J.A., Murphy B.A., Heap S.W. (2005). Alterations in levator ani morphology in elite nulliparous athletes: A pilot study. Aust. N. Z. J. Obstet. Gynaecol..

[4] Kruger J.A., Dietz H.P., Murphy B.A. (2007). Pelvic floor function in elite nulliparous athletes. Ultrasound Obstet. Gynecol..

[5] Borin L.C., Nunes F.R., Guirro E.C. (2013). Assessment of pelvic floor muscle pressure in female athletes. PMR.

[6] Sorrigueta-Hernández A., Padilla-Fernandez B.Y., Marquez-Sanchez M.T., Flores-Fraile M.C., Flores-Fraile J., Moreno-Pascual C., Lorenzo-Gomez A., Garcia-Cenador M.B., Lorenzo-Gomez M.F. (2020). Benefits of physiotherapy on urinary incontinence in high-performance female athletes. Meta-analysis. J. Clin. Med..

[7] Almeida M.B., Barra A.A., Saltiel F., Silva-Filho A.L., Fonseca A.M., Figueiredo E.M. (2016). Urinary incontinence and other pelvic floor dysfunctions in female athletes in Brazil: A cross-sectional study. Scand. J. Med. Sci. Sports.

[8] Hoyte L., Damaser M.S., Warfield S.K., Chukkapalli G., Majumdar A., Choi D.J., Trivedi A., Krysl P. (2008). Quantity and distribution of levator ani stretch during simulated vaginal childbirth. Am. J. Obstet. Gynecol..

[9] Youssef A., Brunelli E., Pilu G., Dietz H.P. (2021). The maternal pelvic floor and labor outcome. Am. J. Obstet. Gynecol. MFM.

[10] Li X., Kruger J.A., Chung J.H., Nash M.P., Nielsen P.M. (2008). Modelling childbirth: Comparing athlete and non-athlete pelvic floor mechanics. Med. Image Comput. Comput. Assist. Interv..

[11] Phipps H., Charlton S., Dietz H.P. (2009). Can antenatal education influence how women push in labour?. Aust. N. Z. J. Obstet. Gynaecol..

[12] Yildirim G., Beji N.K. (2008). Effects of pushing techniques in birth on mother and fetus: A randomized study. Birth.

[13] Wowdzia J.B., McHugh T.L., Thornton J., Sivak A., Mottola M.F., Davenport M.H. (2021). Elite Athletes and Pregnancy Outcomes: A Systematic Review and Meta-analysis. Med. Sci. Sports Exerc..

[14] Bø K., Backe-Hansen K.L. (2007). Do elite athletes experience low back, pelvic girdle and pelvic floor complaints during and after pregnancy?. Scand. J. Med. Sci. Sports.

[15] Sundgot-Borgen J., Sundgot-Borgen C., Myklebust G., Sølvberg N., Torstveit M.K. (2019). Elite athletes get pregnant, have healthy babies and return to sport early postpartum. BMJ Open Sport Exerc. Med..

[16] Sigurdardottir T., Steingrimsdottir T., Geirsson R.T., Halldorsson T.I., Aspelund T., Bø K. (2018). Do female elite athletes experience more complicated childbirth than non-athletes? A case-control study. Br. J. Sports Med..

[17] Wang C., Wei Y., Zhang X., Yue Z., Qianqian X., Yiying S., Shipping S., Li Z., Chunhong L., Yaru F. (2017). Randomized clinical trial of exercise during pregnancy to prevent gestational diabetes mellitus and improve pregnancy outcome in overweight and obese pregnant women. Am. J. Obstet. Gynecol..

[18] Magro-Malosso E.R., Saccone G., Di Mascio D., Berghella V. (2017). Exercise during pregnancy and risk of preterm birth in overweight and obese women: A systematic review and meta-analysis of randomized controlled trials. Acta Obstet. Gynecol. Scand..

[19] Barakat R., Pelaez M., Lopez C., Montejo R., Coteron J. (2012). Exercise during pregnancy reduces the rate of cesarean and instrumental deliveries: Results of a randomized controlled trial. J. Matern. Fetal Neonatal. Med..

[20] Price B.B., Amini S.B., Kappeler K. (2012). Exercise in pregnancy: Effect on fitness and obstetric outcomes-a randomized trial. Med. Sci. Sports Exerc..

[21] Di Mascio D., Magro-Malosso E.R., Saccone G., Marhefka G.D., Berghella V. (2016). Exercise during pregnancy in normal-weight women and risk of preterm birth: A systematic review and meta-analysis of randomized controlled trials. Am. J. Obstet. Gynecol..

[22] Bø K., Hilde G., Staer-Jensen J., Siafarikas F. (2015). Does general exercise training before and during pregnancy influence the pelvic floor “opening” and delivery outcome? A 3D/4D ultrasound study following nulliparous pregnant women from mid-pregnancy to childbirth. Br. J. Sports Med..

[23] Salvesen A.K., Stafne S.N., Torbjørn M., Siv M. (2014). Does regular exercise in pregnancy influence duration of labor? A secondary analysis of a randomized controlled trial. Acta Obstet. Gynecol. Scand..

[24] Berghella V., Saccone G. (2017). Exercise in pregnancy!. Am. J. Obstet. Gynecol..

[25] Bø K., Artal R., Barakat R., Brown W., Dooley M., Evenson K.R., Haakstad L.A.H., Larsen K., Kayser B., Kinnunen T.I. (2016). Exercise and pregnancy in recreational and elite Athletes, 2016 evidence summary from the IOC expert group meeting, Lausanne. Part 2-the effect of exercise on the fetus, labour and birth. Br. J. Sports Med..

[26] Gimovsky A.C. (2021). Defining arrest in the first and second stages of labor. Minerva Obstet. Gynecol..

[27] Swann C., Moran A., Piggott D. (2015). Defining elite athletes: Issues in the study of expert performance in sport psychology. Psychol. Sport Exerc..

[28] Penfield C.A., Wing D.A. (2017). Labor Induction Techniques: Which Is the Best?. Obstet. Gynecol. Clin. N. Am..

[29] Kernberg A., Caughey A.B. (2017). Augmentation of Labor: A Review of Oxytocin Augmentation and Active Management of Labor. Obstet. Gynecol. Clin. N. Am..

[30] Kominiarek M.A., VanVeldhuisen P., Gregory K., Fridman M., Kim H., Hibbard J.U. (2015). Intrapartum cesarean delivery in nulliparas: Risk factors compared by two analytical approaches. J. Perinatol..

[31] Hutchison J., Mahdy H., Hutchison J. (2022). Stages of Labor.

[32] Feeley C., Crossland N., Betran A.P., Weeks A., Downe S., Kingdon C. (2021). Training and expertise in undertaking assisted vaginal delivery (AVD): A mixed methods systematic review of practitioners views and experiences. Reprod. Health.

[33] Goh R., Goh D., Ellepola H. (2018). Perineal tears—A review. Aust. J. Gen. Pract..

[34] American Academy of Pediatrics Committee on Fetus and Newborn, American College of Obstetricians and Gynecologists Committee on Obstetric Practice (2015). The Apgar Score. Pediatrics.

[35] Bewick V., Cheek L., Ball J. (2004). Statistics review 8: Qualitative data—Tests of association. Crit. Care.

[36] Watkins V.Y., O’Donnell C.M., Perez M., Zhao P., England S., Carter E.B., Kelly J.C., Frolova A., Raghuraman N. (2021). The impact of physical activity during pregnancy on labor and delivery. Am. J. Obstet. Gynecol..

[37] Masoud A.T., AbdelGawad M.M., Elshamy N.H., Mohamed O.M., Hashem Z.Y., Abd Eltawab A.K., Samy A., Abbas A.M. (2020). The effect of antenatal exercise on delivery outcomes: A systematic review and meta-analysis of randomized controlled trials. J. Gynecol. Obstet. Hum. Reprod..

[38] Sobhgol S.S., Smith C.A., Dahlen H.G. (2020). The effect of antenatal pelvic floor muscle exercises on labour and birth outcomes: A systematic review and meta-analysis. Int. Urogynecol. J..

[39] Valdes E.G. (2021). Examining Cesarean Delivery Rates by Race: A Population-Based Analysis Using the Robson Ten-Group Classification System. J. Racial. Ethn. Health Disparities.

[40] Blondel B., Alexander S., Bjarnadóttir R.I., Gissler M., Langhoff-Roos J., Novak-Antolič Ž., Prunet C., Zhang W.H., Hindori-Mohangoo A.D., Zeitlin J. (2016). Euro-Peristat Scientific Committee. Variations in rates of severe perineal tears and episiotomies in 20 European countries: A study based on routine national data in Euro-Peristat Project. Acta Obstet. Gynecol. Scand..

[41] Seijmonsbergen-Schermers A.E., van den Akker T., Rydahl E., Beeckman K., Bogaerts A., Binfa L., Frith L., Gross M.M., Misselwitz B., Hálfdánsdóttir B. (2020). Variations in use of childbirth interventions in 13 high-income countries: A multinational cross-sectional study. PLoS Med..

[42] Clesse C., Lighezzolo-Alnot J., De Lavergne S., Hamlin S., Scheffler M. (2018). Statistical trends of episiotomy around the world: Comparative systematic review of changing practices. Health Care Women Int..

[43] Macfarlane A.J., Blondel B., Mohangoo A.D., Cuttini M., Nijhuis J., Novak Z., Ólafsdóttir H.S., Zeitlin J., Euro-Peristat Scientific Committee (2016). Wide differences in mode of delivery within Europe: Risk-stratified analyses of aggregated routine data from the Euro-Peristat study. BJOG.

[44] Sørbye I.K., Oppegaard K.S., Weeks A., Marsdal K., Jacobsen A.F. (2020). Induction of labor and nulliparity: A nationwide clinical practice pilot evaluation. Acta Obstet. Gynecol. Scand..

[45] Austad F.E., Eggebø T.M., Rossen J. (2021). Changes in labor outcomes after implementing structured use of oxytocin augmentation with a 4-hour action line. J. Matern. Fetal Neonatal. Med..

